# Injury surveillance in low-resource settings using Geospatial and Social Web technologies

**DOI:** 10.1186/1476-072X-9-25

**Published:** 2010-05-24

**Authors:** Jonathan Cinnamon, Nadine Schuurman

**Affiliations:** 1 Department of Geography, Simon Fraser University, 8888 University Drive, Burnaby, British Columbia, V5A 1S6 Canada

## Abstract

**Background:**

Extensive public health gains have benefited high-income countries in recent decades, however, citizens of low and middle-income countries (LMIC) have largely not enjoyed the same advancements. This is in part due to the fact that public health data - the foundation for public health advances - are rarely collected in many LMIC. Injury data are particularly scarce in many low-resource settings, despite the huge associated burden of morbidity and mortality. Advances in freely-accessible and easy-to-use information and communication (ICT) technology may provide the impetus for increased public health data collection in settings with limited financial and personnel resources.

**Methods and Results:**

A pilot study was conducted at a hospital in Cape Town, South Africa to assess the utility and feasibility of using free (non-licensed), and easy-to-use Social Web and GeoWeb tools for injury surveillance in low-resource settings. Data entry, geocoding, data exploration, and data visualization were successfully conducted using these technologies, including Google Spreadsheet, Mapalist, BatchGeocode, and Google Earth.

**Conclusion:**

This study examined the potential for Social Web and GeoWeb technologies to contribute to public health data collection and analysis in low-resource settings through an injury surveillance pilot study conducted in Cape Town, South Africa. The success of this study illustrates the great potential for these technologies to be leveraged for public health surveillance in resource-constrained environments, given their ease-of-use and low-cost, and the sharing and collaboration capabilities they afford. The possibilities and potential limitations of these technologies are discussed in relation to the study, and to the field of public health in general.

## Introduction

### Technology and Global Health

Massive gains in health and medicine over the past decades have brought great improvements to quality of life. This is most notable by soaring life expectancy rates and plummeting infant mortality rates; however, these gains have largely been confined to high-income countries [1]. As such, new strategies are required to support health innovation to benefit the low and middle-income countries (LMIC) of the world [2]. One of the current priorities for global health research is information and communication technologies (ICT) [3]. In fact it is argued that advances in ICT will likely have the greatest impact on reaching the Millennium Development Goals [4]. In LMIC where resources for health are severely limited, policy-makers "often have to make difficult decisions that pit investment in new technologies and capacity-building in science and technology against basic population-wide services such as healthcare and water supply and sanitation" [5]. What is required then, are longer-term public health solutions which can be implemented without affecting more immediate necessities. The present study addresses this issue by assessing the possibility for Social Web technologies to support the development of public health surveillance systems for low-resource environments. Social Web technologies may be valuable tools for LMIC because they are designed to be easy to use, and many have no licensing fees. The promise of these technologies was demonstrated through an injury data collection and analysis pilot study conducted in Cape Town, South Africa.

### The Burden of Injury in Low and Middle-Income Countries

The 'invisible epidemic' of injury is one of the leading causes of death in working-aged adults and children in almost every country in the world [6]. Injury is a serious threat to public health and to future generations in all countries around the globe, whether high, middle or low-income [7]. In LMIC however, the problem is particularly acute because of a disproportionately high incidence of injury, a scarcity of resources and prevention efforts, and an extremely low level of funding devoted to this problem in comparison with the high-profile communicable diseases such as HIV/AIDS, tuberculosis, and malaria [8,9]. Of the approximately 5 million deaths annually attributed to injury, 90% occur in LMIC [10,11]. In South Africa, the burden of injury is massive; this huge contribution to overall mortality and morbidity is largely attributed to road-traffic crashes and interpersonal violence [12].

### Injury Surveillance in Low and Middle-Income Countries

In LMIC, high rates of injury are coupled with poor injury surveillance. Public health surveillance involves "ongoing systematic collection, collation, analysis and interpretation of data and the dissemination of information to those who need to know in order that action may be taken" [13]. Injury surveillance systems are relatively well-developed in resource-rich settings; however, they are frequently non-existent in LMIC. This lack of injury data has been highlighted as a major barrier to injury prevention in LMIC [10,14]. It is imperative that data on injury is collected and analyzed so that public health officials can gain a better understanding of the magnitude and characteristics of the problem [9].

### Geographic Information Systems

Geographic Information Systems (GIS) and spatial analysis can play a crucial role in understanding the burden of injury [15,16]. GIS has been used increasingly to help uncover the determinants of injury through analysis of its social and environmental correlates [e.g. [17-20]]. For example, In Kenya, a recent study combined GIS with patient medical records to create an electronic injury surveillance system [14]. This spatially-equipped system was used to ascertain the environmental attributes at the location of injury. GIS technologies range from sophisticated licensed desktop software to free, lightweight, Web-based applications.

### The Social Web and the Geospatial Web

The Social Web (alternatively known as Web 2.0, the read-write Web, etc.) refers to what has been described as the 'second wave' [21] of the World Wide Web. The Social Web represents a paradigm shift from the capabilities of the first incarnation of the Web, despite the original intentions for the Web by its inventor [22]. This shift is identified by a fundamental change in how the Web is used, and by advances in its technological capabilities [23,24]. 'The participatory Web' as opposed to 'Web as information source' is a distinguishing theme of the Social Web, of which user-created content, information-sharing, and collaboration are the hallmarks. User contribution and collaboration aspects of the Social Web have the potential to provide an open platform for political and societal debates, and could increase diversity of opinion, the free flow of information and freedom of expression [25]. The second major theme of the Social Web - a shift in technology usage and capabilities - is characterized by the use of the Internet as a technology platform. Improvements in technology and changing patterns of technology consumption have contributed to the increasing use of no-cost Web-based applications in place of licensed proprietary software. Cheung *et al. *[26] outline the technological innovations that define the Social Web; rich Internet applications, collaboration tools, user contributed content databases, and integrative technologies.

The uptake of rich Internet applications is beginning to take hold as the products offered become more robust. The 'Web office', or Office 2.0 [27] is revolutionizing productivity software availability, with word processing, spreadsheet, and presentation software now available free and accessible anywhere through a Web browser. Collaboration amongst colleagues will become easier, as documents can be stored, edited, and shared online. For example, revisions of a common Web-based document can be undertaken by various authors, while avoiding the problem of having several versions of the document [27]. Google Docs [28] and ThinkFree Office [29] each offer a suite of office productivity tools including word processors, presentation designers, and spreadsheet editors designed to compete with traditional software such as Microsoft Office.

The geospatial Web (or GeoWeb) refers to the "global collection of general services and data that support the use of geographic data in a range of domain applications" [30]. These new technologies, described as "not quite-GIS" by Elwood [31] are bringing Social Web approaches to GIS, thereby democratizing this once exclusive domain [32-35]. Virtual globes such as Google Earth [36], NASA World Wind [37], and ArcGIS Explorer [38] have fast become ubiquitous; much of the success of Google Earth and the other virtual globes stem from their simplicity. This is accomplished by "avoiding reference to the technical details of georeferencing, projections, and figures of the Earth, and presenting the planet as it would appear from a user-controlled viewpoint" [32]. Since GeoWeb platforms are free and easy to navigate, it is expected that the traditional barriers of domain training and financial resources will be reduced, thereby allowing many more people and organizations to leverage geospatial technology for their own purposes.

### Social Web Technology for Health

This new generation Web is poised to initiate great change in the health and science realms [39]. The richer, more complete experience promised to the average Web user extends to researchers, patients, and practitioners in the health and science fields. Despite lagging behind in technology uptake and the use of Social Web applications and services [39-41], health and science stakeholders could gain from this revolution in technology, communication, and interaction. The fact that virtual globes are quick, popular and ubiquitous, render these tools particularly appropriate to use for data visualization [42]. For example, an early demonstration of Google Earth for data visualization in the health domain was a simple interactive application highlighting the Health Authorities in England [43]. A Web site that hosted a Google Earth file was created, which users could download and open on their local machine. Clicking on the geocoded point opened a Web page from the National Health Service within the Google Earth interface with information on that specific health authority. The unique characteristics of the Social Web may be particularly well-suited to LMIC, where lack of finances and trained personnel have traditionally acted as barriers to information and technology uptake [44].

These benefits are already coming to fruition, as Social Web technologies are beginning to appear in support of public health projects in LMIC. Lozano-Fuentes *et al. *[45] describe the use of Google Earth to support the mapping of dengue disease data for visualization and analysis. A dengue decision support system was developed using Google Earth in conjunction with traditional GIS software. Spatial data files for two cities in Mexico were created by tracing satellite imagery using the drawing tools available within the Google Earth software. These tools allowed for the creation of block-level city maps showing the distribution of city blocks with dengue cases. These data layers could then be exported into GIS software for use in a dengue information system. No previous experience with Google Earth was needed to create the spatial data files. Kamadjeu [46] used Google Earth to monitor polio in the vicinity of the Congo River. Google Earth was used to create maps of areas of the river that were unavailable elsewhere. These maps improved public health planning and resource allocation in a region where the topography was previously not fully understood. These recent examples of Social Web software use for public health endeavours represent a major step towards increasing access to technology for LMIC. However, what remains is a true adaptation to the new paradigms of the Social Web, as computer programming and traditional GIS data are still employed in these and most recent examples.

The purpose of this study was to demonstrate the capability of Social Web technologies to be used for public health projects in low-resource settings. As a test case for these technologies, an injury surveillance pilot project was undertaken in Cape Town, South Africa. All aspects of data input, analysis, and visualization were undertaken using Social Web and GeoWeb technologies. An advancement in this study over recent demonstrations was the use of tools that did not require programming or advanced computer skills, GIS data, or licensed proprietary software. This pilot study points to the potential for these technologies to contribute to public health surveillance in low and middle-income countries. The protocols described herein could likely be transferred to other settings and adapted to local capabilities for organizations that wish to engage in public health surveillance.

## Methods

### Needs assessment

A pilot study was conducted in the trauma unit at Groote Schuur Hospital (GSH), a large publicly-funded tertiary hospital in Cape Town. The trauma unit at GSH processes approximately 900 patients monthly, often with serious traumatic injuries largely resulting from interpersonal violence and road-traffic collisions. An initial needs assessment highlighted a need for a streamlined data collection system which could be used for epidemiological analysis and hospital administrative purposes. At the time of the needs assessment, a trauma registry system that consisted of a data collection form and computerized database was in place in the trauma unit, however, it was not capturing the complete population of trauma patients. For the records that were collected in the database, many were incomplete, with vital data missing including the patient's demographic details, the injury cause, the types of injuries sustained, and injury location information. During the needs assessment phase it was hypothesized that the sparsely-populated database was likely to be a result of the current system's complexity, and not a lack of appropriate information available for each patient in the unit. As such, a pilot study was undertaken in order to ascertain the feasibility of developing a trauma registry system which would be able to collate information for all patients seen in the unit. A main element of the pilot study was a 30 day data capture exercise designed to determine the nature of data that could be consistently collected as part of the normal routine of the trauma unit staff. A paper form was developed to collect data on various aspects of the patient, including traditional trauma registry information such as; demographic details, injury type, and injury mechanism. In addition, spatial data were also collected, including details of the patient's residence location, and the location where the injury was sustained. The paper form was modified according to the needs of the proposed data system, and according to the feasibility of field collection. The form was limited to one page in order to keep this duty as streamlined as possible for the busy clinical staff who would be charged with its completion.

The current data management system in place at the hospital was under-used as it was deemed to be too time-consuming and difficult to operate. In this pilot study, we developed and tested a streamlined and readily modifiable trauma data entry and management system using Google Docs [28]. Using the Forms utility within Google Docs, it was possible to create an online data entry form to mimic the paper hardcopy form without the need for programming or advanced computer skills. This online form could be filled out by copying the data from the paper form into the online form. Submitting the form would then populate the spreadsheet with the data without the need to interact with the spreadsheet cells during the data entry phase.

The importance of user-friendly data analysis tools for in-house data exploration and visualization was highlighted during the needs assessment phase. An aspect of the pilot study was to demonstrate the feasibility of free and easy-to-use GeoWeb applications for these purposes in place of complex and costly desktop GIS. Several GeoWeb applications were explored to test their suitability for this purpose, including BatchGeocode, Mapalist, and Google Earth. Ethics approval for this study was granted by Simon Fraser University and the University of Cape Town.

## Results

### Data Collection and Management

**Figure 1:**
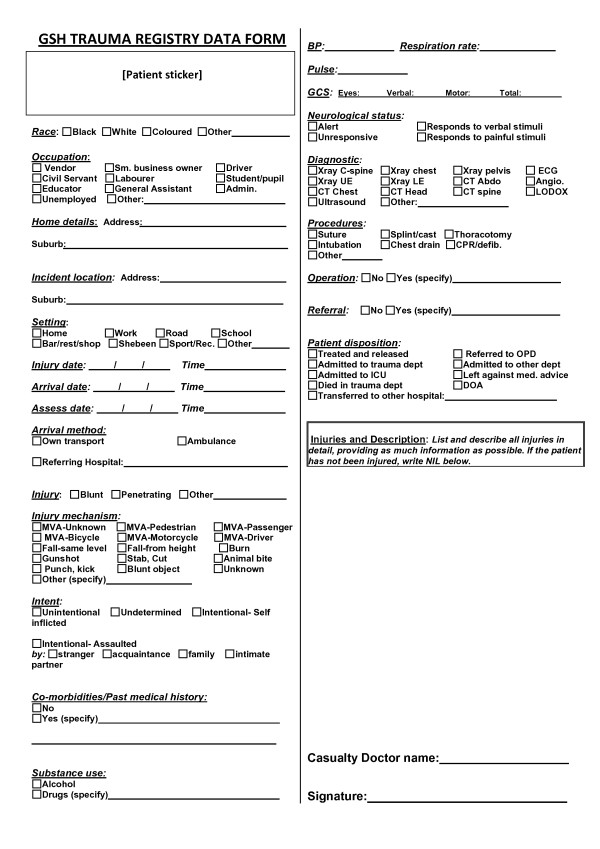
**Paper data collection form**. A one-page paper form was created in order to assemble the data to populate the trauma registry. The form went through several iterations over the course of the pilot study based on the feasibility of collecting the fields. The fields on this final iteration of the form were collectible as part of the regular documentation duties of the trauma unit's clinical staff.

785 patients were recorded in the 30 day data capture feasibility study. Two researchers conducted the data capture; this required assembling the data from various sources and recording it on a one-page paper form, one for each patient. Halfway through the study, doctors and the clinical staff who would eventually be charged with form completion began to assist with completion of the forms as part of their routine patient documentation. These staff were provided training and assistance in order for them to become familiar with the data, where it could be abstracted from, and the purposes of its completion. The form underwent several revisions as the study progressed; the final version of the paper form is shown in Figure 1.

**Figure 2:**
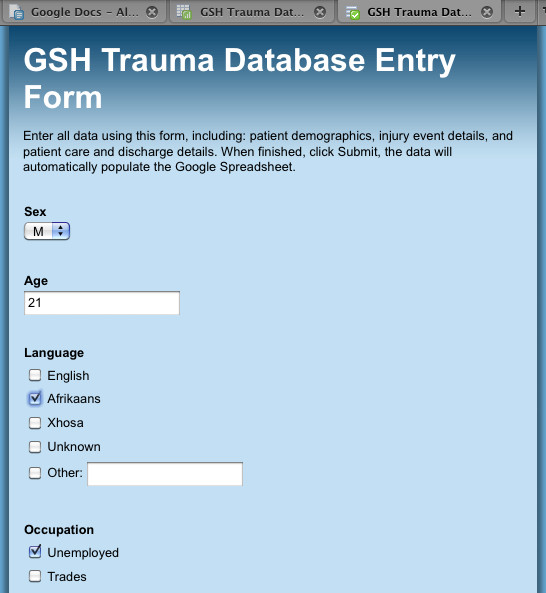
**Google Docs Form used for data entry**. A simple online form was created for entering the injury data into a Google Spreadsheet database. The Form creation utility allows for the design of a data entry system without the need for programming or advanced computer skills. Once completed, the form is submitted and the data automatically populates the spreadsheet.

Between 20 and 50 patients were recorded daily during the data capture study. Each day, one researcher spent between one and three hours entering the data into the trauma database, depending on the number of patients recorded that day. Figure 2 shows the Google Docs Form that was created to allow for easy population of the database, housed online in a security-protected Google Spreadsheet. The online form was designed to allow for a sequential input of data in the same order as on the paper form, i.e. demographic information first, injury event details second, clinical procedures third, and injury details last. As the paper form went through several iterations during the period, the online Google Docs Form had to be redesigned as some fields were removed or had changed position on the paper form. Redesign of the online form was very simple; fields could be removed through a simple deletion, or their sequential position could be changed by dragging the field to its new position and dropping it in its new place.

### Data Exploration and Visualization

**Figure 3:**
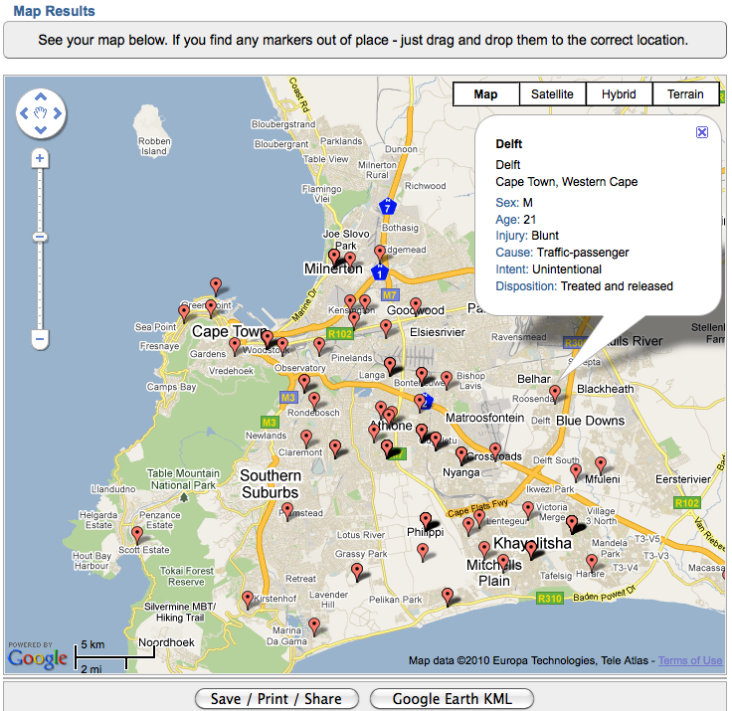
**Screenshot of the BatchGeocode georeferencing system**. BatchGeocode allows the user to paste a table of data into a form, which is then geocoded. Results can be viewed and stored online at www.batchgeocode.com for public or private viewing. In this map, the user can click on a location of interest to see details on the injury event. The map can also be exported as a KML file for further exploration in Google Earth.

A data processing, exploration, and visualization system was developed as a demonstration of the potential for free and simple Social Web and GeoWeb technology to be used for injury control in low-resource settings. Two free Web-based geocoding tools were tested, BatchGeocode http://www.batchgeocode.com, and Mapalist http://www.mapalist.com, both of which operate on the Google Maps platform. BatchGeocode proved to be an easy to use data georeferencing system, with high accuracy at the neighbourhood level in Cape Town. The interface of this Web site allows users to paste a table of data into a form, which is then geocoded (see Figure 3). The updated table complete with geographic coordinates can then be downloaded, or exported in Keyhole Markup Language (KML) format, the file type native to Google Earth. In addition, the results can be saved to a Web page within the site which can be open to public viewing, or restricted. The data can be mapped individually, or it can be grouped into categories by any of the fields in the data table.

**Figure 4:**
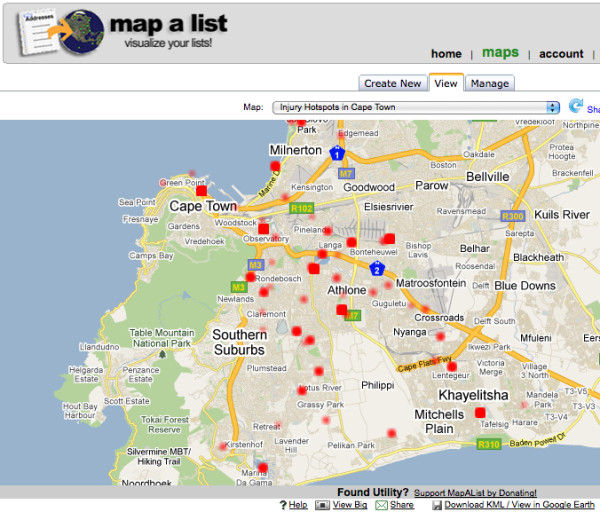
**Mapalist injury concentration (hotspot) map**. A simple hotspot map can be made using the Mapalist georeferencing system. Although the options for hotspot mapping are rudimentary, this is a valuable and distinctive feature of the Mapalist system, as GeoWeb applications generally do not have the capability of visualizing concentrations without API modification. Also of great utility is the option to set the map to update automatically if new data points are added to the linked Google Spreadsheet.

The Mapalist geocoding Web site is designed for beginners; its simple operation is organized as a set of 5 steps that the user proceeds through in order to complete the geocoding, visualization of the data, and saving the work. In Step 1, a Google Spreadsheet (the only type that can be used in Mapalist) is loaded. In Step 2, geocoding parameters are chosen (i.e. the fields with address information are highlighted), and the fields to highlight in pin pop ups are chosen. In Step 3, the data are geocoded according to the address information assigned in the previous step. Step 4 allows the user to configure the resulting output of the data on the map. For example, the data can be mapped individually or grouped by any data value. A unique option available at this stage is the ability to create a simple concentration (hotspot) map, ideal for the purposes of visually highlighting areas with multiple data points, as shown in Figure 4. Additionally, in Step 4 the user can select more advanced options, including displaying only a selection of the data, for example, all patients between the ages of 16-24 who were injured. In Step 5, the user sets the save parameters for the map, including the name, and whether it can be viewed by the public on the Mapalist's Web site, or restricted to private viewing. Also, there is a noteworthy option that allows the mapmaker to set the map to automatically update if the Google Spreadsheet undergoes a modification, such as the addition of new data.

**Figure 5:**
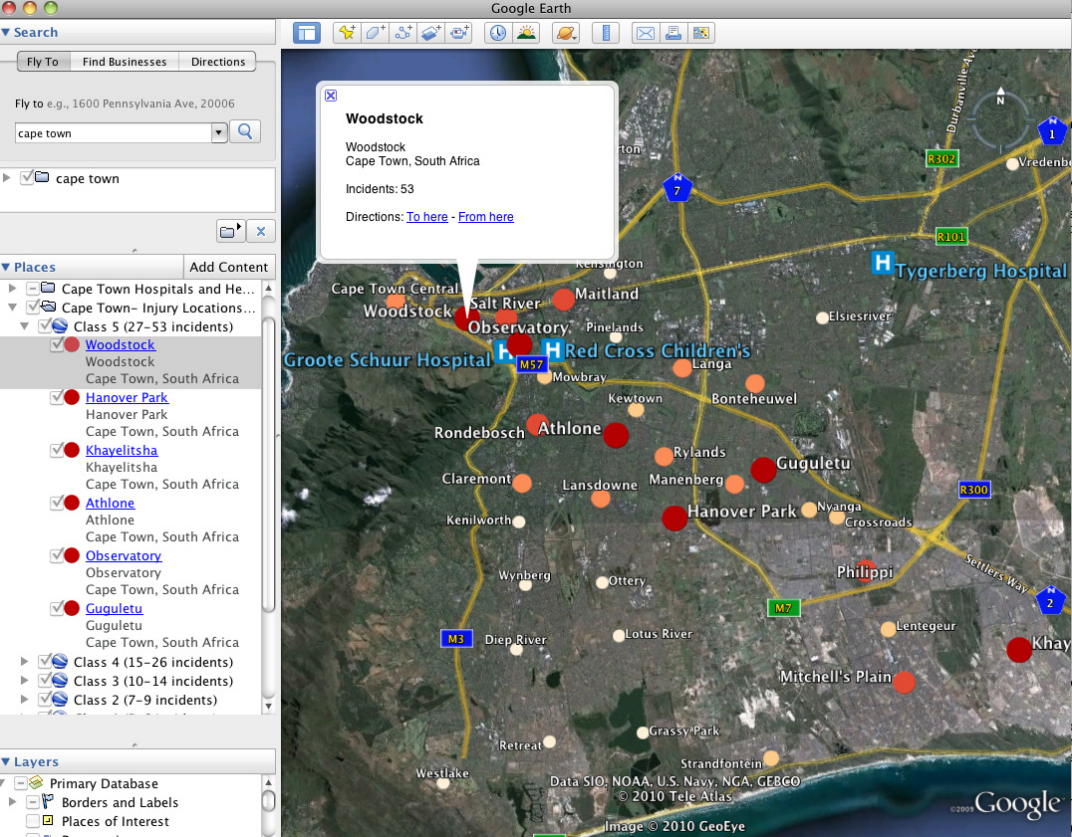
**Google Earth visualization of injuries by suburb**. This may be most useful for epidemiological purposes, as in this visualization the map user can explore the spatial distribution of injury in Cape Town interactively at multiple scales. Incidents were aggregated to the suburb (neighbourhood) level. The visualization was created by exporting the geocoded results from free Web-based geocoding tools as Google Earth (KML) files.

**Figure 6:**
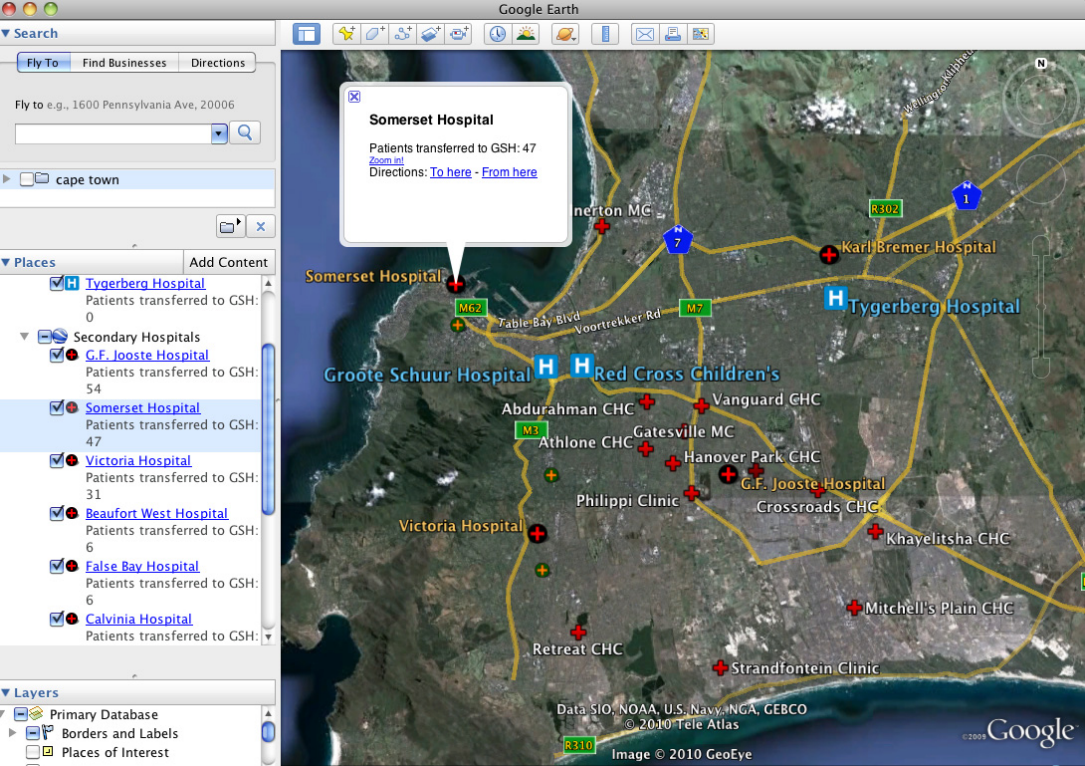
**Google Earth visualization of facilities that transferred patients to GSH**. The map user can use this visualization to observe the number of patients that arrived at GSH from the network of referral hospitals and clinics throughout the city. This visualization may be particularly useful for hospital administration uses, as this information could be used in trauma system planning for the city.

In addition to the visualization options available within the geocoding Web sites, the free version of Google Earth was used to develop a injury data spatial visualization tool. This GeoWeb application was chosen because of its ubiquity as the most popular version of the virtual earth platforms. Two interactive visualizations were created; a map of injury incidents by suburb (Figure 5), and a map of the location of facilities that transferred patients to GSH (Figure 6). In order to create these visualizations, data were aggregated in two new Google Spreadsheets; one for the number of incidents occurring in each suburb, and one for the number of patients transferred to GSH by health care facility. This was a simple process of sorting the data in the appropriate column in the original spreadsheet, summing the number of incidents by suburb and the number of patients transferred by facility, and copying this into the new spreadsheet. KML files for the two visualizations were then created using the online geocoding tools described above. These files could then be opened in Google Earth to allow for exploration and visualization of the spatial data at multiple scales. With these interactive visualizations, the user can easily explore the spatial distribution of injury in Cape Town, and the network of hospitals and health centres in which transferred patients originated from, prior to their arrival at GSH.

### User Evaluations

Informal user-evaluations were conducted throughout the study with hospital staff as a preliminary assessment of the usability and utility of its various components. The participating staff members had no specific background or training in the use of information technologies. The purpose of these evaluations was to assess whether the data collection and analysis system would be useful for the trauma unit, and if the system could be operated and utilized without the need for outside expertise. User-evaluations of the paper form were positive, with clinical staff suggesting that they were easy to complete and were less time-consuming than the current system's two-page form. An informal user-test of the Google Docs Form and Spreadsheets with a non-clinical member of staff who held data entry duties was positive. In comparison with the previous database system, the data entry and management system developed in this study was deemed to be much simpler to use and the records could be entered into the database more rapidly. Once the data had been georeferenced and visualized, members of staff engaged in data exploration within Google Earth. Persons with experience using the platform were easily able to explore the data, and were able to recognize spatial patterns of injury in Cape Town. The users were particularly excited about the potential for the patient transfer facility network visualization to inform future trauma system planning for GSH and the city of Cape Town in general.

## Discussion

In this paper, an injury surveillance pilot study was conducted at a low-resource hospital in Cape Town using Social Web technologies. This pilot study represents the first stage in the development of a sustainable trauma registry that could be used for epidemiological analyses and administrative purposes. Free and easy-to-use data management and analysis tools available on the Web were demonstrated in order to highlight a simple and affordable alternative to traditional software that is expensive and requires comprehensive training. As a result of these benefits, there is great potential for organizations with limited resources to leverage Social Web and GeoWeb technologies for organizing and operationalizing public health surveillance, including for the increasingly recognized problem of injury.

### Social Web Technologies

Technology may hold the key to improved public health in LMIC [47], however, technology advocates must fully understand the barriers to technology uptake faced in these countries. In the case of injury surveillance in LMIC, the two chronic roadblocks to the implementation of sustainable data registries are a lack of finances and trained personnel [48]. This study focused on addressing this problem by designing simple, easy-to-use protocols for collecting, managing, and analyzing injury data.

Social Web technologies may be very attractive options for public health surveillance in settings where resources are limited, most notably as a result of their simplicity and affordability. The technologies demonstrated in this study exemplify simple and affordable data management and visualization solutions. The database that was developed using Google Spreadsheet was simple to set up and operate, including the data entry form and the spreadsheet. In addition, the Forms data entry system was designed to use dropdown menus and tick-boxes and did not require spreadsheet interaction; it is likely that this would result in few data entry errors as opposed to data entry through direct spreadsheet input. With regards to the GeoWeb tools, Mapalist proved to be a better system for the purposes of this study, as it is designed to work specifically with Google Spreadsheets which made the process more streamlined. Additionally, in comparison with the BatchGeocode tools, more visualization options and data query functionality were available.

Virtual globes are largely responsible for introducing geospatial technologies to the masses [49]. Google Earth, the most well-known of the virtual globes is widely used for educational and entertainment purposes, however, its use as a tool for endeavours in science and health is growing [50]. Recent studies have used Google Earth for scientific activities in low-resource settings [e.g. [45,46,50-52]], however in most cases licensed proprietary software was also relied upon in addition to Google Earth, or computer programming was required. In this study, other applications were used to complement Google Earth as described above, however all tools were chosen explicitly because they had no licensing fees, were simple to use, and required no programming or sophisticated computer skills. This is a notable contribution as most organizations in LMIC are unlikely to have access to traditional licensed geospatial software, nor the expertise to operate it. In addition to the abovementioned benefits, there are other reasons that may lead organizations to choose Google Earth. For the average user, Google Earth may be more user-friendly and flexible than traditional GIS, given its more intuitive user-interface that allows the user to readily pan and zoom in for greater detail [33], thereby allowing for exploration of spatial data at multiple scales. Furthermore, organizations may be attracted to Google products since it is developing initiatives to address humanitarian and global health issues through the use of its products, by way of Google Earth Outreach [53] and Google.org [54]. Above all, the ubiquity and simplicity of the Google Earth system make it an ideal platform for data visualization for organizations with financial constraints or no expertise in traditional GIS platforms.

### Information Sharing and User Collaboration

Although this paper is chiefly focused on the technological aspects of the Social Web, another strongly heralded characteristic of the new Web - which is also important to this study - is its enormous potential for sharing and collaboration [55,56]. Through the Social Web's superior sharing and collaboration abilities, and the potential it holds for increased engagement, these visualization and analysis tools could help to bridge the gap between researcher and stakeholder, including policy-makers and citizens [57]. The Google Spreadsheet powered database was accessible and editable anytime, at any Web-enabled computer, providing the user was provided access. For example, as the database was populated by the data entry person, the trauma unit manager could access the most recent records of patients from another computer in real time for administrative purposes, or another data entry person could collaborate on editing the database. The georeferencing Web sites possessed similar sharing and collaboration characteristics. BatchGeocode allowed the user to store their newly created maps on their Web site. These can then be viewed by collaborators on their own computer by providing them with the unique URL that the map is stored on. Mapalist has a more advanced user-account system where users can view, edit, and update all of their maps in an easy-to-use interface. Maps can be privately or publicly viewable; if maps are restricted from public viewing, an email link can be sent to collaborators for viewing on a different computer. The Google Earth platform also sponsors user collaboration and data sharing as KML files of data in Google Earth can be shared instantly via email; this may be one of the most appealing aspects of the system [50]. In fact, the potential for these systems to allow for swift collaboration and sharing may be one of the most promising aspects of the Social Web for use in public health surveillance in any setting, irrespective of the level of resources at hand.

### Future Work

A suitable next step in this study could be to conduct a more thorough user-test of the tools and protocols developed for this study, in order to assess the suitability of the system for local capabilities and to ensure the end result is sustainable. In subsequent phases of this project, attention will be focused on ensuring that the system can be expanded to include other hospitals and jurisdictions. This will require - along with other considerations - the use of international injury coding and database standards. A major direction for future work on this project involves an assessment of the utility of geospatial analysis for injury prevention in the local setting. As GeoWeb tools are not particularly suited to high-level geospatial analysis, it will be necessary to assess the utility of the technology's analysis, visualization, and data exploration capabilities. Although the focus of the present study was on the potential applicability of Social Web tools in this setting, the ultimate goal is to identify environmental and social correlates of injury in Cape Town using the spatial data collected and the visualization tools. Once a sustainable data registry is functioning, a future study will identify high-incident injury locations, and will examine the social and environmental characteristics in order to identify injury risk factors at these locations. This will require obtaining higher-resolution spatial data, which could possibly be obtained by querying the patient or ambulance driver regarding site of injury, followed by a confirmation of the geographic coordinates using global positioning system (GPS) technology. A similar method has been successfully demonstrated in a study by Dwolatzky *et al. *[58] regarding location confirmation in informal settlements of Johannesburg. This is also a focus for future work.

### Limitations and Possibilities

Although the protocols described in this study are likely to be feasible in many middle-income countries such as South Africa, limitations may exist regarding the potential use of Social Web technologies in low-income countries. al-Shobakky & Imsdahl [44] outline several barriers to the uptake of these tools in LMIC, despite their lack of licensing fees and apparent simplicity. First, the computers that exist in some low-resource settings are likely old and may not be able to cope with some of the new Social Web applications. Second, a lack of education and proficiency in English (the language of the Web) will limit who can use them, as these applications are rarely available in local languages. Relying on Web access is an obvious potential limitation; as a result, 'free' technologies are not likely to be without some financial cost. Internet penetration in many of the low and middle-income countries is low. For example, in Africa in 2006, less than 5 out of every 100 people used the Internet, compared with an average of 1 out of every 2 residents of the G8 countries [59]. Google Docs requires Internet access at all times, whereas traditional proprietary spreadsheet applications such as Microsoft Excel do not. This is also the case with the Web-based georeferencing tools. As a result, the sharing and communication benefits of the Web-based tools may be less alluring. Google Earth also requires an Internet connection at the outset of use. However, using Google Earth may less of a hurdle compared with other Social Web tools, as once the satellite images are stored in memory the platform can operate without an Internet connection. These problems pose serious barriers to the uptake of these technologies in low income countries. However, there is some light on the horizon. The cost of Internet access is dropping as advances in wireless technology and hardware are reducing the cost of access [60]. Also, there is a call for new technologies to be developed specifically for low-resource environments, or modifications of existing technologies to allow for access through poor connections [61]. Additionally, there is some evidence that the number of Internet users in developing nations is growing quickly [62]. Despite the constraints, the Social Web holds great promise in LMIC. There is much work to be done however, to ensure that this recent stage in the technological revolution can contribute to the improvement of public health in these settings.

Limitations not specific to LMIC must also be noted as well, such as the potential tendency to over emphasise what is visualized because of the perceived reality of a virtual globe [33]. Proponents of these technologies must be cognisant of the 'wow' factor that these visualization tools can elicit in the viewer. A major issue with the GeoWeb's so-called 'democratization' of GIS is the potential for erroneous mapping and visualization by users unfamiliar with geospatial or epidemiological concepts [63], which could lead to inappropriate decision making. It must also be noted that restrictions may be placed on the use of these Web-based technologies. For example, Google has strict use criteria that determines how its maps and imagery can be used; however, the content can be used for academic purposes such as this study through the 'fair use' guidelines, on condition that attribution is given to Google and its suppliers, and content is identifiable as a Google product [64]. When using Google products or other Web technologies, the user must ensure they are complying with legal terms and conditions. Lastly, relying on these Web-based services for data manipulation and storage may be a point of concern, as potentially sensitive or valuable data could be subject to system instabilities or security breaches. As was done in this study, it is recommended that sensitive health data stored on the Web are anonymized and stored privately.

## Conclusion

Despite considerable advancement in health and medicine in recent decades, the overall health and life-expectancy of citizens in low and middle-income countries remain poor. Public health data are fundamental to public health advances, however current and comprehensive public health data are rare in most LMIC. This is particularly true for the huge problem of injury, given the fact that it is largely overlooked as an immediate health problem, despite its huge toll of morbidity and mortality. As a result, little is known about the magnitude of the problem in many locations, nor its correlates or implications. As health resources are insufficient in LMIC, what is needed are novel solutions for data collection and analysis that will not impinge on already depleted financial and personnel resources. The enormous potential for simple and cost-effective Social Web technologies to be used for injury surveillance in low-resource settings was demonstrated in this study.

Findings of this demonstrative study strongly suggest that the ease-of-use, information sharing, and collaboration aspects of the Social Web may be particularly suited to public health surveillance in low-resource settings such as South Africa, and likely other low and middle-income countries. Although this study was successful in illustrating the potential for new Web technologies, further assessments of the hospital's needs and capabilities will be needed to ensure the system is useful, effective, and sustainable. The results of this study will be useful for organizations that wish to commence public health data collection and analysis in a resource-constrained environment. We encourage researchers and practitioners to continue to examine methods of engaging in data collection and analysis through the use of Social Web, GeoWeb, and related information and communication technologies.

## Competing interests

The authors declare that they have no competing interests.

## Authors' contributions

JC conducted the research and wrote the manuscript. NS conceptualized the paper and edited the manuscript.
